# Phasic Phosphorylation of Caldesmon and ERK 1/2 during Contractions in Human Myometrium

**DOI:** 10.1371/journal.pone.0021542

**Published:** 2011-06-30

**Authors:** Jonathan Paul, Kaushik Maiti, Mark Read, Alexis Hure, Julia Smith, Eng-Cheng Chan, Roger Smith

**Affiliations:** 1 Faculty of Health, Mothers and Babies Research Centre, University of Newcastle, Callaghan, Australia; 2 Hunter Medical Research Institute, John Hunter Hospital, New Lambton, Australia; Brigham and Women's Hospital, United States of America

## Abstract

Human myometrium develops phasic contractions during labor. Phosphorylation of caldesmon (h-CaD) and extracellular signal-regulated kinase 1/2 (ERK 1/2) has been implicated in development of these contractions, however the phospho-regulation of these proteins is yet to be examined during periods of both contraction and relaxation. We hypothesized that protein phosphorylation events are implicated in the phasic nature of myometrial contractions, and aimed to examine h-CaD and ERK 1/2 phosphorylation in myometrium snap frozen at specific stages, including; (1) prior to onset of contractions, (2) at peak contraction and (3) during relaxation. We aimed to compare h-CaD and ERK 1/2 phosphorylation *in vitro* against results from *in vivo* studies that compared not-in-labor (NIL) and laboring (L) myometrium. Comparison of NIL (n = 8) and L (n = 8) myometrium revealed a 2-fold increase in h-CaD phosphorylation (ser-789; *P* = 0.012) during onset of labor *in vivo*, and was associated with significantly up-regulated ERK2 expression (*P* = 0.022), however no change in ERK2 phosphorylation was observed (*P* = 0.475). During *in vitro* studies (n = 5), transition from non-contracting tissue to tissue at peak contraction was associated with increased phosphorylation of both h-CaD and ERK 1/2. Furthermore, tissue preserved at relaxation phase exhibited diminished levels of h-CaD and ERK 1/2 phosphorylation compared to tissue preserved at peak contraction, thereby producing a phasic phosphorylation profile for h-CaD and ERK 1/2. h-CaD and ERK 1/2 are phosphorylated during myometrial contractions, however their phospho-regulation is dynamic, in that h-CaD and ERK 1/2 are phosphorylated and dephosphorylated in phase with contraction and relaxation respectively. Comparisons of NIL and L tissue are at risk of failing to detect these changes, as L samples are not necessarily preserved in the midst of an active contraction.

## Introduction

Human myometrial contractions last approximately 60 seconds and are followed by extended periods of relaxation. Whilst protein phosphorylation cascades have been linked to myometrial contractility, little is known about the regulation of these phosphorylation events during the periods of relaxation.

Within human myometrium, evidence exists linking the phosphorylation of high molecular weight caldesmon (h-CaD) and extracellular signal-regulated kinase 1/2 (ERK 1/2) to the generation of contractions [1], [2], [3], [4], [5], [6]. As an essential regulator of smooth muscle contractility [1], [2], h-CaD functions to inhibit myosin ATPase activity [7], [8] as well as interact with tropomyosin [9], [10], [11] to block the binding site of myosin on actin. Together these two properties prevent actin-myosin cross bridge cycling, which is critical to the generation of contractile force [3], [4]. h-CaD also possesses binding domains for calcium (Ca^2+^)-calmodulin [12], and recently significantly increased myometrial h-CaD expression at ≥37 weeks gestation (relative to non-pregnant myometrium) was correlated with decreased Ca^2+^ sensitivity, suggesting that h-CaD is a potent inhibitor of Ca^2+^-induced smooth muscle contraction [6]. Phosphorylation of h-CaD on serine residue 789, which is a known ERK phosphorylation site [5], induces conformational changes that relieve inhibition of myosin ATPase as well as exposing the myosin binding site on actin. Together these changes permit actin-myosin cross bridge cycling and the subsequent generation of contractile force. Li et al., demonstrated that h-CaD is phosphorylated in human myometrial strips following the application of stretch [6]. These studies reported that gestational stretch activates focal adhesion signaling via focal adhesion kinase (FAK), which, acting through smooth muscle Archvillin (smAV), recruits ERK to dense plaques, leading to ERK activation and subsequent phosphorylation of h-CaD on Ser789 [6]. Li et al., also examined h-CaD phosphorylation *in vivo*, and although the authors reported significantly increased h-CaD phosphorylation in term laboring myometrium, statistical significance was achieved relative to non-pregnant myometrium, with no significant difference evident between term non-laboring and term laboring human myometrium.

Given that contraction associated proteins such as h-CaD are regulated by protein phosphorylation, we hypothesised that rapid protein phosphorylation events may be implicated in the phasic nature of spontaneous myometrial contractions. We aimed to test this hypothesis using the novel approach of snap freezing myometrial tissue at specific stages during the development of contractions, including prior to the onset of contractions, at the peak of contraction and during periods of relaxation between contractions. Here we report the first evidence of phasic phospho-regulation of h-CaD and ERK 1/2 in parallel to phasic contractions in human myometrium *in vitro*. Furthermore, we provide the first evidence that h-CaD phosphorylation is increased during the late gestation transition from term, not-in-labor (NIL) myometrium to term, laboring (L) myometrium, and that this transition is associated with increased ERK expression.

## Materials and Methods

### Sample acquisition

These studies were approved by the Hunter and New England Area Research Committee, adhering to guidelines of the University of Newcastle and John Hunter Hospital, Newcastle, Australia. All participants gave informed written consent. Human myometrial samples (5×5×10 mm) were obtained from the lower uterine segment of term singleton pregnancies undergoing cesarean section. Age of the participants averaged 31.75±2.06 years for NIL studies and 29±1.76 years for L studies (age comparison; p = 0.164). For NIL studies myometrial biopsies were collected from women ranging from 38–39 weeks gestation (38.87±0.125 weeks, mean ± SEM), whilst for L studies gestation ranged from 36–42 weeks (39.8±0.66 weeks, mean ± SEM)(gestation comparison; p = 0.09). Subjects underwent elective cesarean section prior to the onset of labor (NIL cohort) or emergency cesarean section after the onset of labor (L cohort). Reasons for elective cesarean section were previous cesarean section (5 samples), 3^rd^/4^th^ degree tear (2 samples) or placental praevia (1 samples), whilst reasons for emergency cesarean section were non-reassuring cardiotocograph (fetal distress; 3 samples), undiagnosed breach (2 samples), failed forceps delivery (1 sample), poor progress (1 sample) or herpes simplex virus (1 sample). Following delivery of the placenta, all women were administered 5 units of syntocinon directly into an intravenous line as part of standard care for the prevention of post-partum hemorrhage. Myometrial biopsies were then excised 3–5 min after administration of the syntocinon. All samples were therefore exposed to oxytocin however exposure was brief (3–5 min). In addition, two samples from the L cohort also received 10 units of syntocinon during the induction of labor. After biopsy, myometrial samples were dissected from connective tissue and either frozen in liquid nitrogen or placed into ice-cold saline and stored at 4°C (maximum 16 h) for contractility studies.

### Isometric tension recordings (myometrial bioassay)

Myometrial samples from non-laboring women were cut into strips (7×2×2 mm) and suspended in organ baths containing 30 ml Krebs-Henseleit buffer with 1.89 mM CaCl_2_. Strips were connected to a Grass FT03C force transducer (Grass Instruments, Quincy, MA) and 1 g passive tension applied. Buffer was replaced five times during the first hour, with strips re-tensioned to 1 g passive tension following each wash. Thereafter strips were maintained at 37°C (pH 7.4) and continuously bubbled with 95% O_2_/5% CO_2_ until spontaneous rhythmic contractions developed. Data were digitized using a Maclab8E data-acquisition system and contraction status visualised in real time using Chart software (ADI, Melbourne, Australia). Strips were closely monitored until reaching appropriate contraction phases and then snap frozen using liquid nitrogen (i) after 15 mins of washing but prior to the onset of any contractions, (ii) at maximum contraction once rhythmic contractions were established, and (iii) at maximum relaxation between rhythmic contractions. Snap freezing was performed by rapidly raising the tissue strips out of the organ bath and cutting the threads tethering the strips to the transducer. The strips were collected into 5 ml tubes that were immediately capped and plunged into liquid nitrogen. Total time for the snap freezing procedure was 5–7 seconds. The tissue was then stored at −80°C.

### Protein extraction and one-dimensional (1D) SDS-PAGE

Frozen tissue was pulverized under liquid N_2_ and 100 mg tissue homogenized in 1 ml lysis buffer (7 M urea, 2 M thiourea, 4% CHAPS, 30 mM Tris) supplemented with PhosSTOP phosphatase inhibitor (Roche, Castle Hill, NSW) and Complete Mini Protease Inhibitor (Roche). The protein extraction homogenate was incubated at 4°C for 30 mins on a rotary mixer before being centrifuged at 11,000× *g*, for 15 min at 4°C. Supernatant was collected and protein content determined by 2D Quant Kit (GE Healthcare, Piscataway, NJ). Reagents and equipment were obtained from Invitrogen (Mt. Waverley, Australia), unless stated otherwise. Protein extracts (20 µg per lane) were loaded onto 4–12% NuPAGE gels and separated using a Novex Mini-Cell system at constant voltage (200 V for 50 min; Invitrogen). For ‘NIL versus L’ studies, myometrial biopsies were pulverized and extracted from eight non-laboring women and eight laboring women. Samples were run across two gels with 4 non-laboring samples, 4 laboring samples and a calibrator sample were included on each gel.

### Pro-Q Diamond Phosphoprotein Labeling

Following SDS-PAGE separation, proteins were labeled with Pro-Q® Diamond Phosphoprotein Gel Stain (Invitrogen) according to the manufacturer's standard instructions for staining minigels. Pro-Q® Diamond Staining was visualised using a FujiFilm LAS-3000 Luminescent Image Analyser. Following visualisation of Pro-Q® Diamond labeling, gels were immediately stained with SYPRO® Ruby Protein Gel Stain (Invitrogen) according to the manufacturers standard procedure for minigel staining. SYPRO® Ruby protein staining was imaged on a FujiFilm LAS-3000 Luminescent Image Analyser. To ensure specific phosphoprotein labeling was achieved, a Peppermint™ Stick molecular weight marker consisting of 2 phosphoproteins and 4 non-phosphorylated proteins was included on all gels.

### Immunoblotting

After 1D SDS-PAGE, proteins were transferred to Hybond-C nitrocellulose (Amersham Biosciences, Buckinghamshire, UK) using the XCell II Blot Module (Invitrogen). For anti-phosphotyrosine visualization, blots were first stained with SYPRO® Ruby Protein Blot stain (Invitrogen) according to the manufacturers instructions. During immunoblotting all incubations were performed on a rocking platform. Membranes were blocked in 5% skim milk in TBS/T (500 mM NaCl, 20 mM Tris, 0.01% Tween-20) overnight at 4°C. Blocking solution was decanted and primary antibody applied at previously optimised dilutions in 10 mL 1% skim milk in TBS/T for 2 h at RT. Antibodies against phospho-h-CaD (cat.# 07-156, Cell Signaling, Lake Placid, NY), total-h-CaD (cat.# MAB3576, Millipore, Billerica, MA), phospho-ERK 1/2 (cat.# 4377S, Cell Signaling), total-ERK 1/2 (cat.# 4695, Cell Signaling) and phospho-MLC (cat.# sc-19849-R, Santa Cruz Biotechnology, Santa Cruz, CA) were applied at 1∶2000 dilution, whilst antibodies against phospho-tyrosine (cat.# 03-7720, Invitrogen) and α-smooth muscle actin (α-SMA) (cat.# A5228, Sigma-Aldrich, Castle Hill, NSW) were applied at 1∶2500 and 1∶10,000 respectively. Blots were subjected to 4×5 min washes with 100 mL of TBS/T. Washed blots were then incubated in horseradish peroxidase (HRP)-conjugated anti-rabbit IgG (cat.# 7074, Cell Signaling) or anti-mouse IgG (cat.# 7076, Cell Signaling) secondary antibody as appropriate. Secondary antibodies were applied at 1∶2500 dilution in 10 mL of 1% skim milk in TBS/T for 1 h at RT. Blots were washed for 3×5 min in 100 mL of TBS/T before immunoreactive products were detected with enhanced chemiluminescence (ECL Western blotting reagents; GE Biosciences, Buckinghamshire, UK) and visualized using the Fuji Intelligent Dark Box LAS-3000 Image Reader (Fuji Photo Film, Tokyo, Japan). To ensure internal controls, blots were first probed with the phospho-specific antibodies before being stripped and re-probed with antibodies against the total proteins. Membranes were stripped by 2×4 min incubations in 100 mL of 0.2 M NaOH. Stripped blots were then washed for 3×3 min in TBS/T before being re-probed according to the outlined regime. Phosphorylation of MLC was examined as a proof of concept.

### Densitometry and Statistics

Densitometric analysis of immunoreactive protein bands was performed using Multigauge software (Fuji Photo Film) supplied with the LAS-3000. Where necessary, optical density (OD; arbitrary units) was normalized between blots using the incorporated calibrator sample on each blot (NIL versus L studies). OD was expressed as a percentage of the phosphorylated target proteins relative to the total protein density. Similarly, OD of total h-CaD and total ERK 1/2 was expressed as a percentage of α-SMA OD, which was used as a loading control given that α-SMA abundance in myometrium remains unchanged throughout pregnancy [13]. Statistical analysis was conducted using Stata 9.2 software (StataCorp, College Station, TX).

The distribution of each outcome variable was assessed using a histogram and the skewness/kurtosis test for normality. The ratio of phospho-h-CaD to total h-CaD was skewed right, hence median and interquartile ranges have been reported. The ratio of total h-CaD/SMA, p-ERK 1/2/total ERK 1/2, and total ERK 1/2/SMA were normally distributed, hence mean and standard deviation are reported. Statistical comparisons were undertaken comparing the NIL (n = 8) and L (n = 8) group. Two-sample t-tests with equal variance were performed for the normally distributed variables and the non-parametric equivalent, Mann-Whitney U test, was performed for the ratio of phospho-h-CaD to total h-CaD. Statistical analyses were carried out using Intercooled Stata 11 (StataCorp LP, College Station, Texas, USA). For all tests, *P*≤0.05 was considered significant.

## Results

### h-CaD and ERK expression in non-laboring and laboring human myometrium

**Figure 1:**
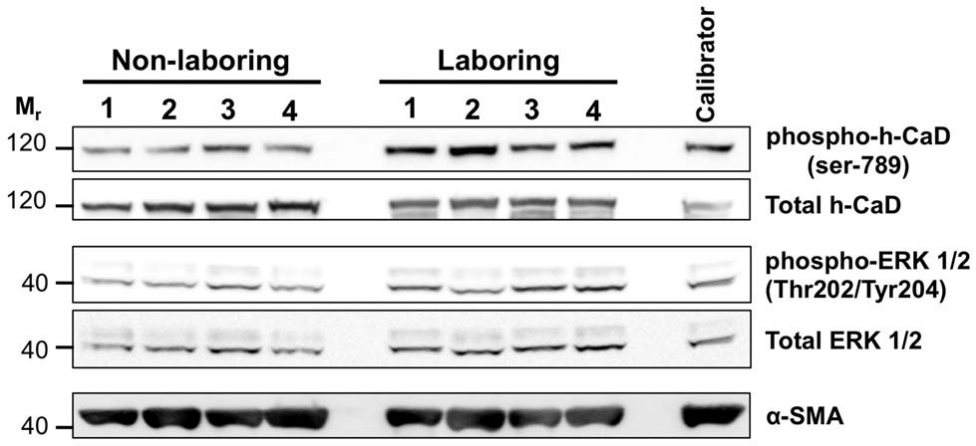
h-CaD and ERK 1/2 expression in NIL and L term human myometrium. NIL and L myometrial protein extracts were separated by SDS-PAGE and western transferred onto nitrocellulose membrane. Membranes were probed with antibodies against phospho-h-CaD (1∶2000), total h-CaD (1∶2000), phospho-ERK 1/2 (1∶2000), total ERK 1/2 (1∶2000) and α-SMA (1∶10000). A total 8×NIL and 8×L samples were analysed. Representative image demonstrates detection of these proteins on a single blot containing 4×NIL and 4×L myometrial samples, as well as a calibrator sample that was included to allow densitometric comparison across separate blots. M_r_ = relative molecular mass×1000.

**Figure 2:**
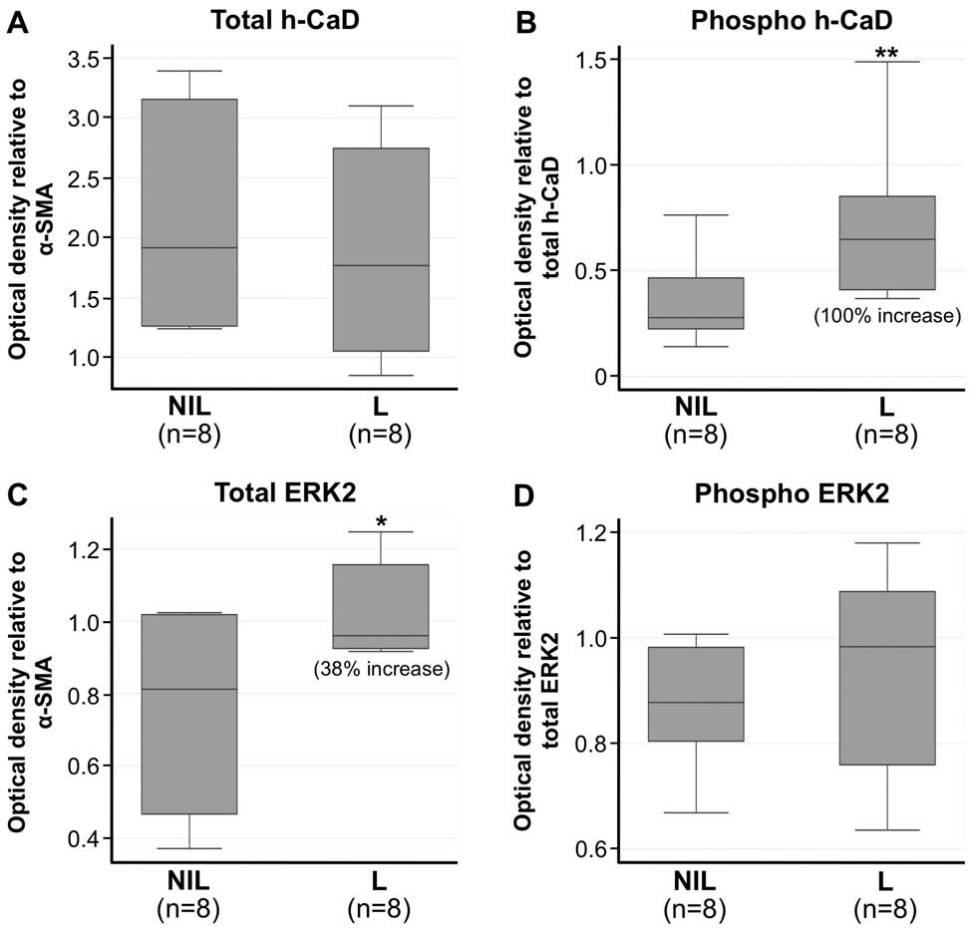
Densitometric analysis of h-CaD and ERK2 expression in NIL and L myometrium. Densitometric analysis was performed for 8×NIL and 8×L myometrial samples following immunodetection of phospho-h-CaD, total h-CaD, phospho-ERK 1/2, total ERK 1/2 and α-SMA. (A) Onset of labor was associated with a statistically significant, 2-fold up-regulation in h-CaD phosphorylation, whilst (B) total h-CaD expression remained unchanged. (C) ERK2 phosphorylation remained unchanged following the onset of labor, however (D) total ERK2 expression underwent a statistically significant 1.4-fold up-regulation in association with the onset of labor. *p<0.05, **p<0.02.

h-CaD expression and phosphorylation were examined in 8×NIL and 8×L term pregnant myometrial samples via western blot (Figure 1). Upon densitometric analysis we observed no significant difference in total h-CaD expression between NIL and L myometrium (*P* = 0.567; Figure 2A). Analysis of h-CaD phosphorylation revealed a two-fold increase of serine-789 phosphorylation following the onset of labor *in vivo* (*P* = 0.012, Figure 2B). We also examined ERK 1/2 expression and phosphorylation in these samples (Figure 1) given that serine-789 is a known ERK phosphorylation site [5]. ERK1 protein was undetectable. ERK2 expression was detectible and densitometric analyses revealed that the onset of labor was associated with a small yet statistically significant increase (38%) of ERK2 expression (*P* = 0.022; Figure 2C). Although total ERK2 expression increased by 38% during the onset of labor, densitometric analysis revealed no change in the ratio of phospho-ERK2/total ERK2 between NIL and L laboring human myometrium (*P* = 0.475; Figure 2D).

### Phasic phosphorylation during myometrial contractions *in vitro*

**Figure 3:**
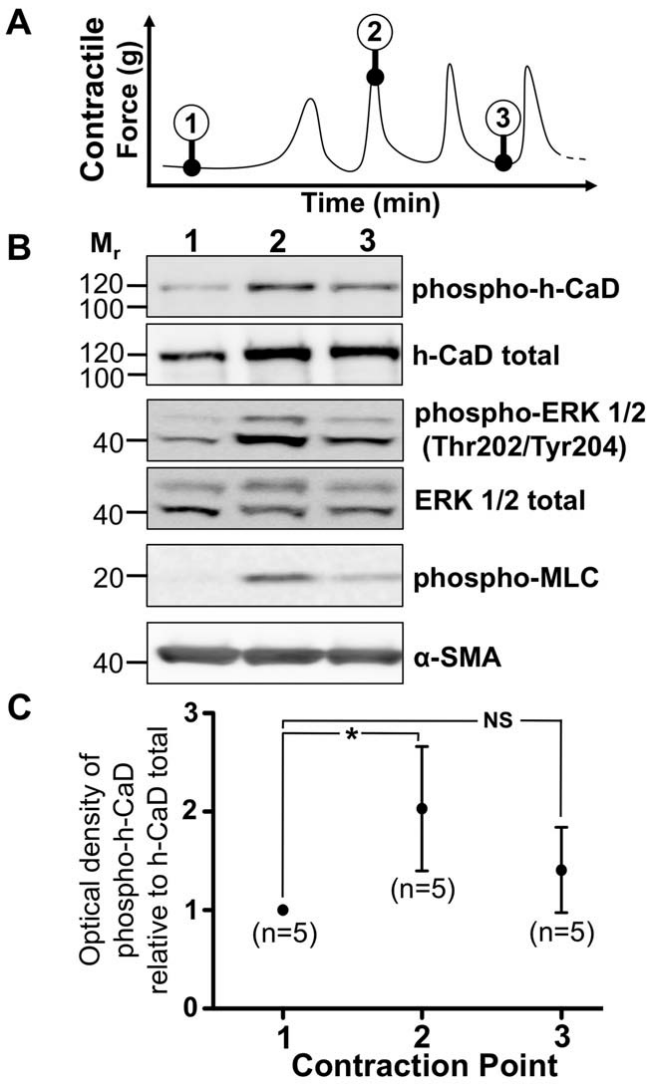
h-CaD and ERK 1/2 phosphorylation during myometrial contractions *in vitro*. (A) Myometrial strips were snap frozen at specific stages during the development of contractions. The tissue was then pulverised and subjected to protein extraction. Protein (20 µg) was separated by 1D PAGE and transferred to a nitrocellulose membrane. (B) Membranes were probed with antibodies against phospho-h-CaD (1∶2000), total h-CaD (1∶2000), phospho-ERK 1/2 (1∶2000) and total ERK 1/2 (1∶2000), as well as α-SMA (1∶10000), representative images shown of 5 replicates. (C) Non-parametric analyses of the non-normalised raw data revealed a statistically significant 2-fold increase in h-CaD phosphorylation during the transition from point 1 to point 2. No significant difference was observed between contraction points 1 and 3 or 2 and 3. *p<0.05. M_r_ = relative molecular mass×1000.

In addition to investigating the regulation of h-CaD phosphorylation during the onset of labor *in vivo*, we examined h-CaD phosphorylation during the development of contractions *in vitro*. We obtained myometrial biopsies at caesarean section from women at term. These biopsies were dissected into strips and the strips suspended in organ baths under tension. We then snap froze the myometrial strips at specific stages during the development of spontaneous contractions, including (1) prior to the onset of any contractions, (2) at peak contraction and (3) during relaxation between individual contractions (see Figure 3A). As a proof of concept, we analysed myosin light chain (MLC) phosphorylation (serine-19) at these specific stages during the development of contractions. We observed that non-contracting myometrium exhibited minimal levels of MLC phosphorylation. The transition from non-contractile myometrium at point 1 to peak contraction at point 2 however was associated with extensive up-regulation of MLC phosphorylation (Figure 3B). Furthermore, the transition from peak contraction at point 2 to relaxation at point 3 was associated with a reduction of MLC phosphorylation, demonstrating that within our model MLC is phosphorylated and de-phosphorylated in phase with contractions. Having demonstrated the efficacy of our model we proceeded to investigate h-CaD expression during the establishment of contractions *in vitro*. Consistent with our *in vivo* ‘NIL versus L’ data, the establishment of spontaneous contractions *in vitro* was associated with increased phosphorylation of h-CaD (Figure 3B). When normalised against total h-CaD protein, densitometric analysis of five replicates revealed that the transition from non-contractile tissue at point 1 to peak contraction at point 2 was associated with an average two-fold increase in h-CaD phosphorylation (*P* = 0.043, Figure 3C). Furthermore, the transition from peak contraction at point 2 to relaxed tissue at point 3 was associated with a reduction in h-CaD phosphorylation. The transition from point 2 to point 3 corresponds to 5–8 min of temporal spacing, and during this time h-CaD phosphorylation declined to levels that were not significantly elevated above that of tissue preserved at point 1 (*P* = 0.138; Figure 3C).

During *in vitro* studies we also examined ERK 1/2 phosphorylation as the potential regulator of h-CaD phosphorylation. ERK1 detection was minimal however ERK2 was highly expressed in human myometrial samples utilised for the *in vitro* studies. Where our comparison of NIL and L myometrium failed to show a difference in ERK2 activation, our *in vitro* analyses demonstrated that the onset of contractions was associated with increased ERK2 phosphorylation. Figure 3B clearly illustrates that the transition from non-contractile tissue (point 1) to tissue at peak contraction (point 2) is associated with an extensive up-regulation of ERK2 phosphorylation. Furthermore, the transition from peak contraction (point 2) to relaxation (point 3) was associated with a decline in ERK2 phosphorylation (Figure 3B). ERK2 phosphorylation therefore increased during the transition from non-contractile tissue to tissue at peak contraction, and then declined during the transition from peak contraction to relaxation. Although ERK1 expression was substantially less then ERK2, ERK1 exhibited the same pattern of protein phosphorylation in phase with onset and subsidence of contractions. This phasic pattern is consistent with our findings on h-CaD phosphorylation.

### Phosphoprotein labeling in human myometrium

**Figure 4:**
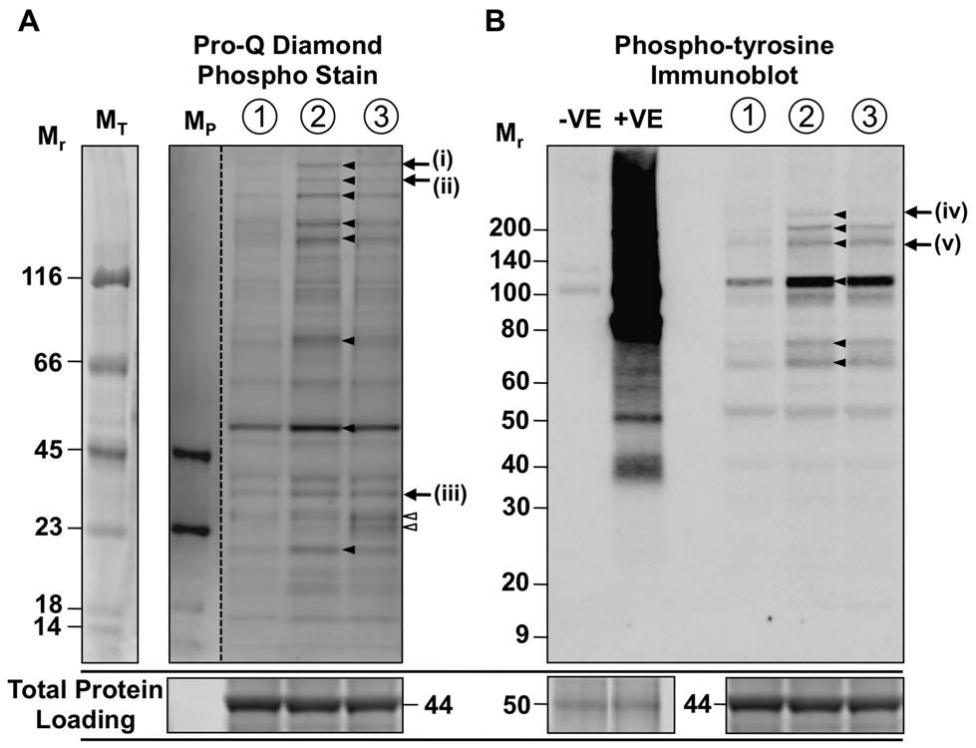
Protein phosphorylation associated with myometrial contractility. Myometrial strips were snap frozen at specific stages during the development of contractions. The tissue was then pulverised, protein extracted and separated by 1D SDS-PAGE. (A) Phosphorylated myometrial proteins were labelled in gel with Pro-Q® Diamond Phospho Stain and visualised using a FujiFilm LAS-3000 luminescent image analyser. The gel was then SYPRO® Ruby Protein stained to visualise total protein and ascertain equal loading. Lanes M_P_ and M_T_ illustrate the Peppermint™ Stick molecular weight marker labelled with Pro-Q® Diamond Stain and SYPRO Ruby Protein Stain respectively. Dotted line indicates removal on none-relevant gel lanes. (B) Separated proteins were western transferred to nitrocellulose membrane. Total protein loading was visualised using SYPRO Ruby Blot Stain and equal loading across lanes confirmed. Membranes were then probed with mouse anti-phosphotyrosine (1∶2500) and anti-mouse IgG-HRP (1∶2500). The immunoreactive bands were detected using a FujiFilm LAS-3000 luminescent image analyser. Positive and negative controls were protein extracts from capacitated and non-capacitated human spermatozoa respectively. Black arrowheads and white arrowheads indicate phosphorylation events associated with contraction and relaxation respectively. (i), (ii) and (iv) highlight bands exhibiting phasic phosphorylation, whilst phosphorylation remained increased for bands (iii) and (v). Representative images shown from 3 replicates. M_r_ = relative molecular mass×1000. M_T_ = molecular weight marker stained for total protein detection, M_P_ = molecular weight marker stained for phospho protein detection.

We sought to visualise global changes in protein phosphorylation associated with the development of contractions *in vitro*. Specific labeling of phosphoproteins with Pro-Q® Diamond Phosphoprotein Gel Stain was confirmed using the Peppermint stick marker included on all gels, as outlined in methods. Pro-Q® Diamond staining of myometrial proteins from contraction phases 1, 2 and 3 revealed that the development of contractions *in vitro* was associated with increased phosphorylation of a cohort of proteins. Phosphoproteins that underwent prominent phospho-changes during the transition from non-contractile tissue to tissue at peak contraction are indicated by the black arrowheads (Figure 4A). The major phosphoprotein band observed had a M_r_ of ∼50×10^3^, however at least 5 high molecular weight proteins (M_r_>100×10^3^) and a protein with M_r_ of ∼80×10^3^ were also observed to undergo phospho regulation. A series of other phosphoprotein bands also exhibited increased phosphorylation although to varying lesser extents. The transition from peak contraction at point 2 to relaxation at point 3 was associated with reduced phosphorylation of multiple proteins. This was particularly evident for the two high molecular weight phosphoprotein bands labeled (i) and (ii), and is consistent with the phasic phosphorylation profiles observed for h-CaD and ERK 1/2. In contrast, other proteins were phosphorylated at peak contraction and remained phosphorylated despite the myometrium having transitioned into relaxation, such as phosphoprotein band (iii). Two proteins with M_r_ of 23×10^3^ and 25×10^3^ (indicated by white arrowheads) exhibited increased phosphorylation during the transition from peak contraction at point 2 to relaxation at point 3. The identities and characterisation of these protein bands is the subject of further investigation.

Our complementary analyses into the specific detection of tyrosine phosphorylation events yielded similar results (Figure 4B). Immunoblot detection of phospho-tyrosine residues demonstrated that the transition from non-contractile tissue at point 1 to tissue at peak contraction at point 2 was associated with increased tyrosine phosphorylation of at least six myometrial proteins. Of these the major phospho-tyrosine protein band observed had a M_r_ of ∼115×10^3^. As per the Pro-Q Diamond staining, we again observed that the transition from peak contraction at point 2 to relaxation at point 3 was associated with reduced tyrosine phosphorylation of specific proteins, such as band (iv) for example, whilst tyrosine phosphorylation of other proteins, such as band (v), remained high despite the transition into relaxation. These phospho-tyrosine protein bands are now being characterized.

## Discussion

At labor the human uterus develops co-ordinated regular contractions that force the fetus through a softened cervix to achieve delivery. Previous studies of human myometrium have compared tissue obtained from women prior to the onset of labor with samples taken after the onset of labor; yet even during labor the uterus is relaxed for the majority of time. Here we have developed and demonstrated a method for studying the molecular events associated with contraction and relaxation in term myometrial tissue.

Li et al. 2009 have previously reported that h-CaD expression is significantly up-regulated in human myometrium at ≥37 weeks gestation [6]. Statistical significance was however achieved relative to non-pregnant tissue as opposed to term NIL myometrium. In the presently reported studies we compared term NIL myometrium against term L myometrium and have provided the first evidence that h-CaD phosphorylation is increased during this late gestation transition *in vivo* (Figure 2B). Increased phosphorylation of h-CaD during labor is consistent with its role as a phosphorylation-regulated actin binding protein and regulator of smooth muscle contraction [1], [2], [5], [7], [8]. With no further elaboration on these ‘NIL versus L’ results, one could assume that h-CaD phosphorylation remains increased for the duration of labor. Data obtained from our *in vitro* model however indicates that this is not actually the case. We took the novel approach of snap-freezing non-laboring myometrial strips at specific stages during their transition from non-contractile tissue through to tissue engaged in rhythmic contractions that arise spontaneously once myometrium is suspended under tension (in organ baths). Unlike ordinary tension assays or ‘NIL versus L’ comparisons, we sub-divided our contractile samples into those preserved at peak contraction and those preserved at relaxation. This approach provides additional insight into changes that occur during the transition from peak contraction to intervals of relaxation that exist in between individual contractions. Our data on MLC provides ‘proof of concept’ for this approach, as MLC was phosphorylated in tissue preserved at peak contraction, yet minimally dephosphorylated in non-contractile tissue or tissue preserved during relaxation (Figure 3B).

Our studies reveal that phosphorylation of h-CaD parallels the onset and subsidence of contractions. That is, h-CaD is phosphorylated at the peak of myometrial contraction, and de-phosphorylated during periods of relaxation between individual contractions. This pattern of phasic phosphorylation was also observed for ERK 1/2, and provides indirect evidence that ERK 1/2 regulates the phasic phosphorylation of h-CaD on serine-789 during myometrial contractions *in vitro*, which is consistent with serine-789 being a known ERK phosphorylation site [5].

Results of our global phosphorylation analyses demonstrate that ERK 1/2 and h-CaD are just two members of a cohort of proteins that exhibit phasic phosphorylation during myometrial contractions. We visualised at least 14 phosphorylation events associated with the development of contractions. A sub-set of these proteins underwent dephosphorylation during relaxation, while others remained phosphorylated.

Our evidence of phasic phosphorylation in parallel to contractions is of key significance as it demonstrates that phasic phosphorylation events may be missed by traditional experimental approaches. For instance, during our ‘NIL versus L’ analyses we observed increased h-CaD phosphorylation during the onset of labor, as well as significantly up-regulated expression of ERK2 (Figure 2). Examination of ERK2 phosphorylation however revealed no change during labor (Figure 2D), despite contrasting *in vitro* evidence demonstrating that ERK 1/2 is phosphorylated at peak contraction (Figure 3B). The inability of the ‘NIL versus L’ comparison to identify changes in ERK 1/2 phosphorylation is attributed to the fact that laboring samples are inevitably a heterogeneous group. That is, although all the myometrial samples from our L cohort were obtained from women classified clinically as ‘in labor’, samples are not necessarily excised and preserved in the midst of an active contraction. In fact, given a typical situation where individual contractions are shorter then the interval between contractions, it is actually more likely that laboring samples are preserved during relaxation as opposed to contraction. As our data have shown, significant differences in protein phosphorylation and thus regulation exist between these two contraction states that must be taken into account during experimental design and interpretation of results. So whilst comparing NIL myometrium against L myometrium does indeed have the capacity to highlight some contraction-associated changes in the myometrium, such as h-CaD phosphorylation, other changes are far more transient and as such may only be captured at very specific stages of contraction, such as ERK 1/2 phosphorylation. Recognizing the limitations of the ‘NIL versus L’ approach raises obvious questions as to whether phasic myometrial phosphorylation events could actually be measured *in vivo*, and if so how could it be achieved?

This investigation provides a unique insight into phasic phosphorylation events that occur during myometrial contractions in humans. We have demonstrated that h-CaD phosphorylation increases late in gestation during the onset of labor *in vivo*. Venturing beyond this, we further demonstrate that although h-CaD is phosphorylated during the development of contractions, the regulation of serine-789 phosphorylation is actually phasic, in that h-CaD is once again de-phosphorylated during periods of relaxation. We have shown that in humans the transition from term, NIL myometrium to term L myometrium is associated with up-regulated ERK2 expression, and that during the development of spontaneous contractions *in vitro* ERK 1/2 demonstrates the same phasic phosphorylation exhibited by h-CaD. We demonstrate that h-CaD and ERK 1/2 are just two members of a cohort of proteins subjected to this type of phasic regulation, and we reveal that the transient nature of these phosphorylation events impedes our ability to capture evidence of their existence using traditional experimental approaches. Finally, where protein phosphorylation status is of concern, we stress the importance of considering the marked changes that exists between contraction and relaxation phases of human myometrium during experimental design and interpretation of results.

## References

[1] Marston SB, Redwood CS (1991). The molecular anatomy of caldesmon.. Biochem J.

[2] Sobue K, Sellers JR (1991). Caldesmon, a novel regulatory protein in smooth muscle and nonmuscle actomyosin systems.. J Biol Chem.

[3] Morgan KG, Gangopadhyay SS (2001). Invited review: cross-bridge regulation by thin filament-associated proteins.. J Appl Physiol.

[4] Huang R, Li L, Guo H, Wang CL (2003). Caldesmon binding to actin is regulated by calmodulin and phosphorylation via different mechanisms.. Biochemistry.

[5] D'Angelo G, Graceffa P, Wang CA, Wrangle J, Adam LP (1999). Mammal-specific, ERK-dependent, caldesmon phosphorylation in smooth muscle. Quantitation using novel anti-phosphopeptide antibodies.. J Biol Chem.

[6] Li Y, Reznichenko M, Tribe RM, Hess PE, Taggart M (2009). Stretch activates human myometrium via ERK, caldesmon and focal adhesion signaling.. PLoS One.

[7] Sadovsky Y, Friedman SA (1992). Fetal fibronectin and preterm labor [letter; comment].. N Engl J Med.

[8] Szpacenko A, Dabrowska R (1986). Functional domain of caldesmon.. FEBS Lett.

[9] Fujii T, Ozawa J, Ogoma Y, Kondo Y (1988). Interaction between chicken gizzard caldesmon and tropomyosin.. J Biochem.

[10] Graceffa P (1987). Evidence for interaction between smooth muscle tropomyosin and caldesmon.. FEBS Lett.

[11] Horiuchi KY, Chacko S (1988). Interaction between caldesmon and tropomyosin in the presence and absence of smooth muscle actin.. Biochemistry.

[12] Sobue K, Muramoto Y, Fujita M, Kakiuchi S (1981). Purification of a calmodulin-binding protein from chicken gizzard that interacts with F-actin.. Proc Natl Acad Sci U S A.

[13] Ewoane C, Cavaille F (1990). Interaction of actin from pregnant and nonpregnant human uterus with the uterine myosin subfragment-1: actin activated ATPase activities and actin binding.. Biochem Life Sci Adv.

